# miR-1301-3p Promotes Cell Proliferation and Facilitates Cell Cycle Progression *via* Targeting SIRT1 in Gastric Cancer

**DOI:** 10.3389/fonc.2021.664242

**Published:** 2021-04-27

**Authors:** Dakui Luo, Hao Fan, Xiang Ma, Chao Yang, Yu He, Yugang Ge, Mingkun Jiang, Zekuan Xu, Li Yang

**Affiliations:** Department of General Surgery, The First Affiliated Hospital of Nanjing Medical University, Nanjing, China

**Keywords:** miR-1301-3p, gastric cancer, cell proliferation, cell cycle, SIRT1

## Abstract

So far, many existing evidences indicate that microRNAs (miRNA) are closely associated with the tumorigenesis and progression of various tumors. It has been reported that miR-1301-3p is abnormally expressed in several malignant tumors. However, the role of miR-1301-3p in gastric cancer (GC) remains unclear and is worth studying. Through qRT-PCR, the expression of miR-1301-3p and SIRT1 were detected in GC tissues and cells. The cell proliferation and cell cycle were measured through CCK-8 assay and clone formation assay. Dual luciferase reporter assay was used to determine the target of miR-1301-3p. Though tumorigenesis assay, we monitored the effect of miR-1301-3p on GC cell growth *in vivo*. miR-1301-3p was upregulated in GC tissues and cells in our study. Overexpression of miR-1301-3p accelerated GC cell proliferation, cell cycle progression and tumorigenesis. Notably, altering the expression miR-1301-3p caused deregulation of Cyclin D1, CDK4, c-Myc and P21. Furthermore, SIRT1 was the direct target of miR-1301-3p by luciferase reporter assay. After transfecting with miR-1301-3p inhibitor, we found that knockdown of SIRT1 could enhance the ability of proliferation. Our results identify miR-1301-3p as a novel potential therapeutic target that is associated with the tumorigenesis and progression of gastric cancer.

## Introduction

Gastric cancer (GC) is the most common malignant tumor and one of the leading causes of tumor-related death (1). The absence of obvious symptoms in the early period of GC results in dismal prognosis (2). Therefore, identification of novel crucial biomarkers seems to be urgent.

MicroRNAs (miRNAs) are a class of short non-coding RNAs that function by binding to the 3’-UTR of a target gene, leading to their inhibition in post-transcriptional level or degradation (3, 4). Differential miRNAs expression has been reported in enormous oncologic studies (5–7). We lucubrated the global miRNA expression profiles in GC derived from The Cancer Genome Atlas (TCGA) and identified miR-1301-3p was up-regulated significantly in GC. Previous studies have shown that microRNA-1301-3p inhibits the invasion and migration of HepG2 cells (8). However, not long after the discovery, others proved that miR-1301-3p is highly expressed and promotes tumorigenesis in liver cancer cells (9). A latest study demonstrated that miR-1301 could inhibit biological function of hepatocellular carcinoma cells, such as the migration, invasion, and so on (10). The paradoxical results veiled the mysterious functions of miR-1301-3p, making the researches focused on its role in cancers more fascinating. Besides that, the correlations between miR-1301-3p and prostate cancer progression as well as glioma were revealed, and miR-1301-3p may act as oncogene that accelerate the process of prostate carcinogenesis through targeting PPP2R2C or tumor suppressor gene inhibiting the proliferation of glioma cells (11, 12). These results prompt us to further study the role of miR-1301-3p in GC.

SIRT1, a class III histone deacetylase that depends on nicotinamide, could regulate the physiological and pathological process by creating proteins and DNA methylation (13, 14), playing dual effects on the development of neoplasms (15–18). A previous study found that SIRT1 could result in G1-phase arrest through NF-kB/Cyclin D1 signaling, retarding proliferation of GC cells (19). As a result, SIRT1 could affect tumorigenesis by regulating cell cycle progression.

In this study, we initially evaluated miR-1301-3p expression and the biologic process in GC. Determining SIRT1 as the direct target of miR-1301-3p by a series of assays. In miR-1301-3p-inhibited GC cells, further knockdown of SIRT1 could reverse the inhibitory effect of miR-1301-3p knockdown on gastric cancer cell function. Our results identify miR-1301-3p as a novel potential therapeutic target that is associated with the tumorigenesis and progression of GC.

## Materials and Methods

### Gastric Cancer Tissues and Cells

GC and normal tissues from sixty patients were collected from 2010 to 2015 in the First Affiliated Hospital of Nanjing Medical University. All cases were diagnosed as primary gastric cancer and no neoadjuvant chemotherapy or radiotherapy was received. The ethics committee of the First Affiliated Hospital of Nanjing Medical University has approved the study. All patients received informed consent.

All Human GC cell lines in this research were obtained from Cell Center of Shanghai Institutes for Biological Science. The above cell lines were cultured in RPMI-1640 medium (Gibco, USA) by mixing with 10% fetal calf serum (WISENT, Canada) under humidified conditions of 5% CO_2_ at 37 °C, respectively. HEK-293T cell line was cultivated in DMEM medium (Gibco, USA) blended with 10% fetal bovine serum at 37°C in a humidified condition which contains 5% CO_2_.

### Quantitative Real-Time PCR

Total RNA was extracted by using Trizol reagent (Invitrogen, Carlsbad, CA, USA). After that, for detecting miR-1301-3p expression level, each RNA sample was polyadenylated in the presence of ATP using polyA polymerase, then the decorated RNA was mixed with polyT adaptor: 5’-GCGAGCACAGAATTAATACGACTCACTATAGGTTTTTTTTTTTT-3’, eventually, cDNA was obtained by using RevertAid First Strand cDNA Synthesis Kit (Thermo, USA). For quantify SIRT1 mRNA expression, cDNA from RNA samples were contained by using Primescript RT reagent (Takara, Otsu, Japan). Reverse transcription real-time polymerase chain reaction (RT-PCR) was performed using SYBR Green Master Mix (Vazyme, Nanjing, China) in a Steponeplus instrument (Applied Biosystems, Foster City, CA, USA). The primers involved in this study are shown in **Table 1**. The amplification reaction was carried out in a volume of 10 μL, including 5 μL of the master mix, 0.2 μL of the primer, and 100 ng of cDNA. The reaction procedure was set to 95 °C for 5 minutes, then 40 cycles, 95 °C for 10 seconds, 60 °C for 30 seconds. All procedures are performed in triplicate.

**Table 1:** Sequences used in this study.

Gene	Primer sequence
hsa-miR-1301-3p-F	5’-TTGCAGCTGCCTGGGAGTGACTTC-3’
U6-F	5’-ATTGGAACGATACAGAGAAGATT-3’
U-primer	5’-GCGAGCACAGAATTAATACGAC-3’
adaptor	5’-GCGAGCACAGAATTAATACGACTCACTATAGGTTTTTTTTTTTT-3’
SIRT1-F	5’-TAGCCTTGTCAGATAAGGAAGGA-3’
SIRT1-R	5’-ACAGCTTCACAGTCAACTTTGT-3’
β-actin-F	5’-CTTGCAGCTCTTCCGGAGTC-3’
β-actin-R	5’-GCTCAGTGAGCATCAGCGTG-3’

### Western Blotting

The total protein from GC cells has suffered different management. Protein was separated on SDS-PAGE and transferred to PVDF membranes. Then, blocking it and probing the membranes overnight at 4°C with primary antibodies: rabbit anti-human Cyclin D1, rabbit anti-human c-Myc, rabbit anti-human P21, rabbit anti-human β-actin (dilution 1:1000, Cell Signaling Technology, Massachusetts, USA), mouse anti-human CDK4 and rabbit anti-human SIRT1(dilution 1:2000, Abcam, Cambridge, UK). After that, probing the membranes with the HRR-conjugated anti-mouse or anti-rabbit IgG antibodies (dulation 1:20000, Jackson Immunoresearch) at room temperature for 2 hours.

### Flow Cytometry

Fixing transfected cells overnight in 75% iced ethanol. Thereafter, the cells were cultured with propidium iodide (PI) staining solution for 30 minutes, then, analyzing the cell distribution by flow cytometry (BD Biosciences).

### Cell Transfection

In present study, MGC-803 and SGC-7901 cells were treated with miR-1301 inhibitor, miR-1301 mimics and corresponding negative controls using lentivirus (GenePharma, Shanghai, China). Small interfering (Si) SIRT1 and normal control oligonucleotides (GenePharma, Shanghai, China) were transfected by using Lipofectamine 3000 Transfection Reagents (Invitrogen, CA, USA). We confirmed the transfection efficiency with qRT-PCR and western blot.

### Luciferase Reporter Assay

We predicted the possible miR-1301-3p binding sites in SIRT1 3’ untranslated region (3’-UTR) by TargetMiner, microRNA.org, TarBase. Sequences corresponding to the 3’-UTR of SIRT1 mRNA and containing the mutated or wild-type miR-1301-3p binding sequence were designed and processed by GeneScript (Nanjing, China). To generate the SIRT1 3’-UTR reporter constructs (pMIR-WT-SIRT1 and pMIR-MUT-SIRT1), Hind III and Spe I restriction enzymes were used to digest the pMIR-report plasmid. The synthesized WT and Mut sequences were then connected to the Spe I/Hind III sites of the pMIR-report plasmid respectively (Applied Biosystems). We co-transfected H293T cells with miR-1301-3p mimics or control, pRL-TK vector, and pMIR-REPORT vector using Lipofectamine 2000 (Invitrogen) in 24-well plates. After 48 hours of transfection, the luciferase activity was detected by using double luciferase reporting and analysis system (Promega).

### CCK-8 Assay

1000 cells were seeded and cultured with 100ul RPMI-1640 medium containing 10% FBS in 96-well plates. At the designated time point of each day, investigating the GC cell proliferation using cell counting kit-8 (CCK-8). In general, replacing the medium by 110 ul RPMI-1640 with 10 ul CCK-8, after that, incubating the cells for 2 hours and measuring the cell viability.

### Colony Formation Assay

500 GC cells were seeded and incubated for about 14 days in 6-well plates while the obvious colony was observed. Then, the plates were washed and stained with PBS and crystal violet, respectively. Counting the colonies number by Photoshop software. Each clone contains more than 50 cells with a size of 0.3-1.0 mm.

### Immunohistochemistry (IHC)

Fixing cancerous and paracancerous tissue specimens by using 4% formalin, and the specimens were then embedded in paraffin. After the endogenous protein and peroxide were blocked, in order to specifically detect SIRT1 (Abcam, Cambridge, UK), a 4 μm thick section was incubated with the primary antibody overnight at 4°C. The sections were washed with PBS next day, and co-incubated with the HRP-polymer conjugated secondary antibody for 1 hour at 37°C. Next, 3,3-diaminobenzidine solution was used to stain sections for 3 minutes and hematoxylin was utilized to counterstain the nuclei. A blinded manner was utilized to examine the sections. Three fields were randomly selected for each section to determine the intensity of cell staining and percentage of positive tumors.

### Tumor Xenograft in Nude Mice

The Institutional Animal Care and Use Committee of Nanjing Medical University approve all animal experiments. A total of thirty BALB/c nude mice (4 weeks old) were purchased from the Animal Center of Nanjing Medical University. The number of 2*10^6^ cells blended with 200 ul PBS was inoculated subcutaneously into each flank of nude mice. We evaluate the tumors with calipers every four days. Three weeks later, mice were euthanized. We calculate the tumor volume with the formula of “volume = (width^2^ × length)/2”.

### Statistical Analysis

All data were presented as mean ± deviation. Student’s t test (two-tailed) was used to perform the statistical analyses with SPSS 22.0. χ^2^ test was used to analyze the categorical data. P<0.05 was considered statistically significant.

## Results

### miR-1301-3p Is Overexpressed in Both GC Tissues and Cells

**Figure 1:**
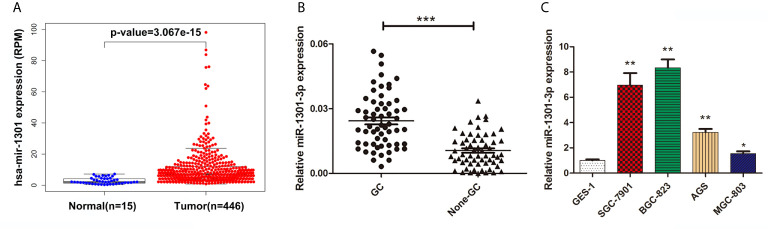
Expression of miR-1301 in human gastric tissues and cells. **(A)** The expression levels of miR-1301 in TCGA database. **(B)** The expression levels of miR-1301-3p in 60 pairs of GC and adjacent normal tissues. **(C)** The expression levels of miR-1301-3p in GES-1 and GC cells. *p < 0.05, **p < 0.01, ***p < 0.001.

To begin with, we performed a comprehensive analysis of The Cancer Genome Atlas (TCGA) data, miR-1301 was found up-regulated at GC tissue level (**Figure 1A**). To validate this result, the expression of miR-1301-3p in GC and normal tissues was detected by qRT-PCR method, which showed that miR-1301-3p was upregulated in GC (**Figure 1B**). Similarly, miR-1301-3p expression was significantly increased in GC cell lines compared to GES-1 cells (**Figure 1C**).

### miR-1301-3p Promotes the Proliferation and Accelerates Cell Cycle Progression of GC Cells

**Figure 2:**
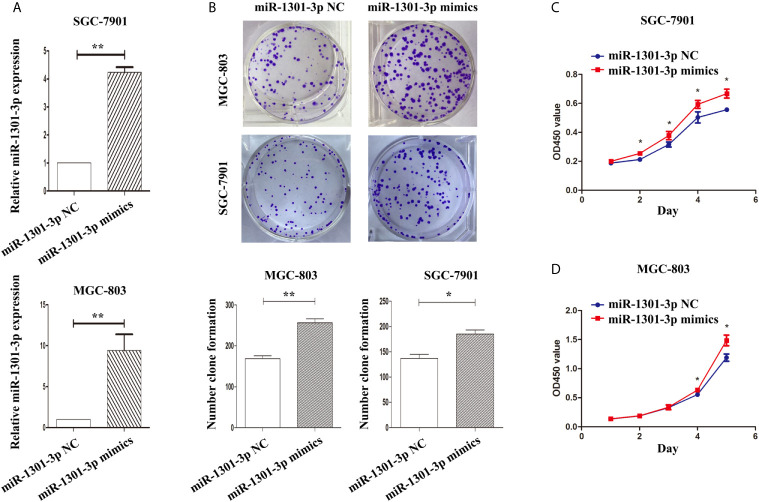
Overexpression of miR-1301-3 promotes the proliferation of GC cells. **(A)** qRT-PCR was utilized to verify the efficacy of transfection. **(B)** Colony formation assay demonstrated overexpression of miR-1301-3p enhanced the proliferation of GC cells. **(C, D)** CCK-8 assay demonstrated that overexpression of miR-1301-3p promoted the proliferation of GC cells. *p < 0.05, **p < 0.01.

**Figure 3:**
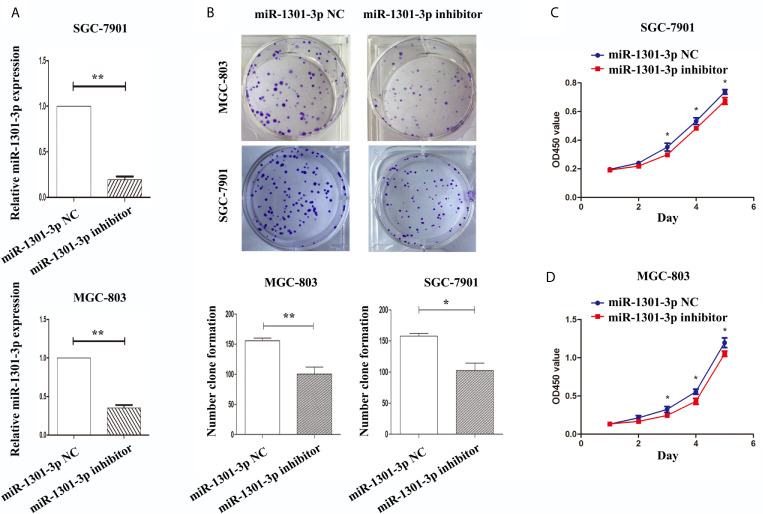
Knockdown of miR-1301-3p inhibited the proliferation of GC cells. **(A)** qRT-PCR was utilized to verify the efficacy of transfection. **(B)** Colony formation assay demonstrated knockdown of miR-1301-3p inhibited the proliferation of GC cells. **(C, D)** CCK-8 assay demonstrated that knockdown of miR-1301-3p inhibited the proliferation of GC cells. *p < 0.05, **p < 0.01.

**Figure 4:**
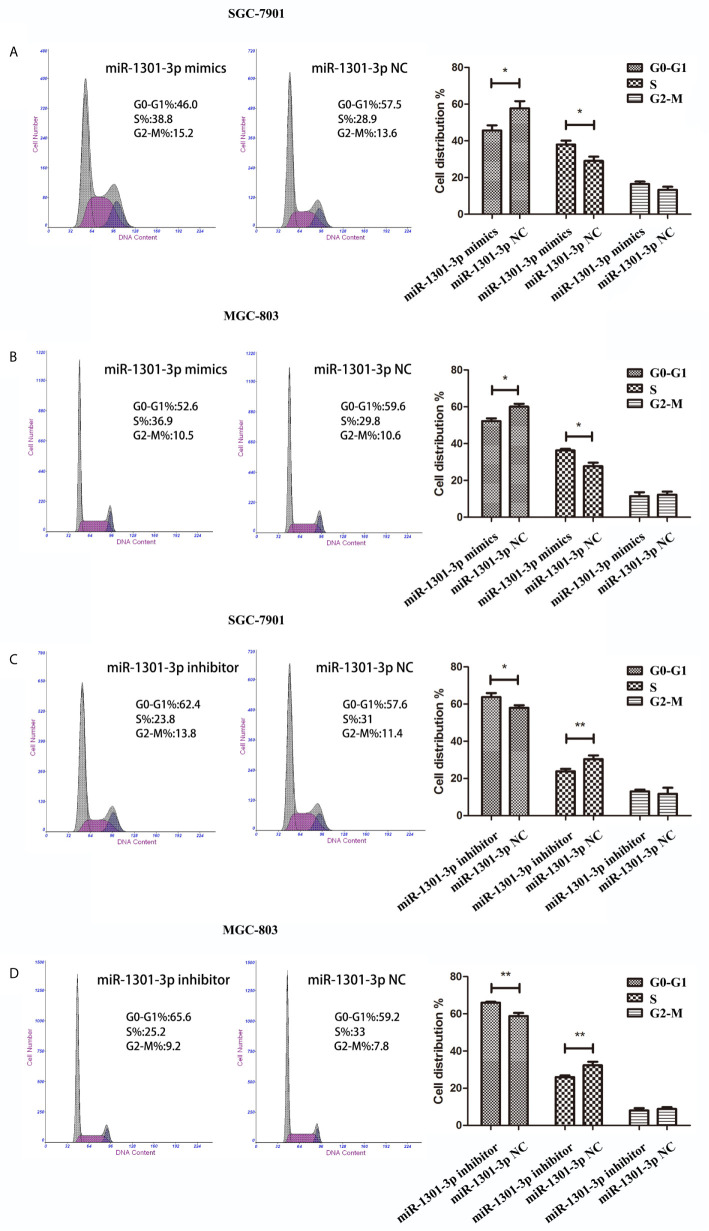
miR-1301-3p facilitated cell cycle progression. **(A, B)** miR-1301-3p mimics resulted in a decrease of the proportion in the G0/G1 phase and an increase of the proportion in the S phase compared with the control. **(C, D)** miR-1301-3p inhibitor increased the proportion in the G0/G1 phase and decreased the proportion in the S phase compared with the control. *p < 0.05, **p < 0.01.

**Figure 5:**
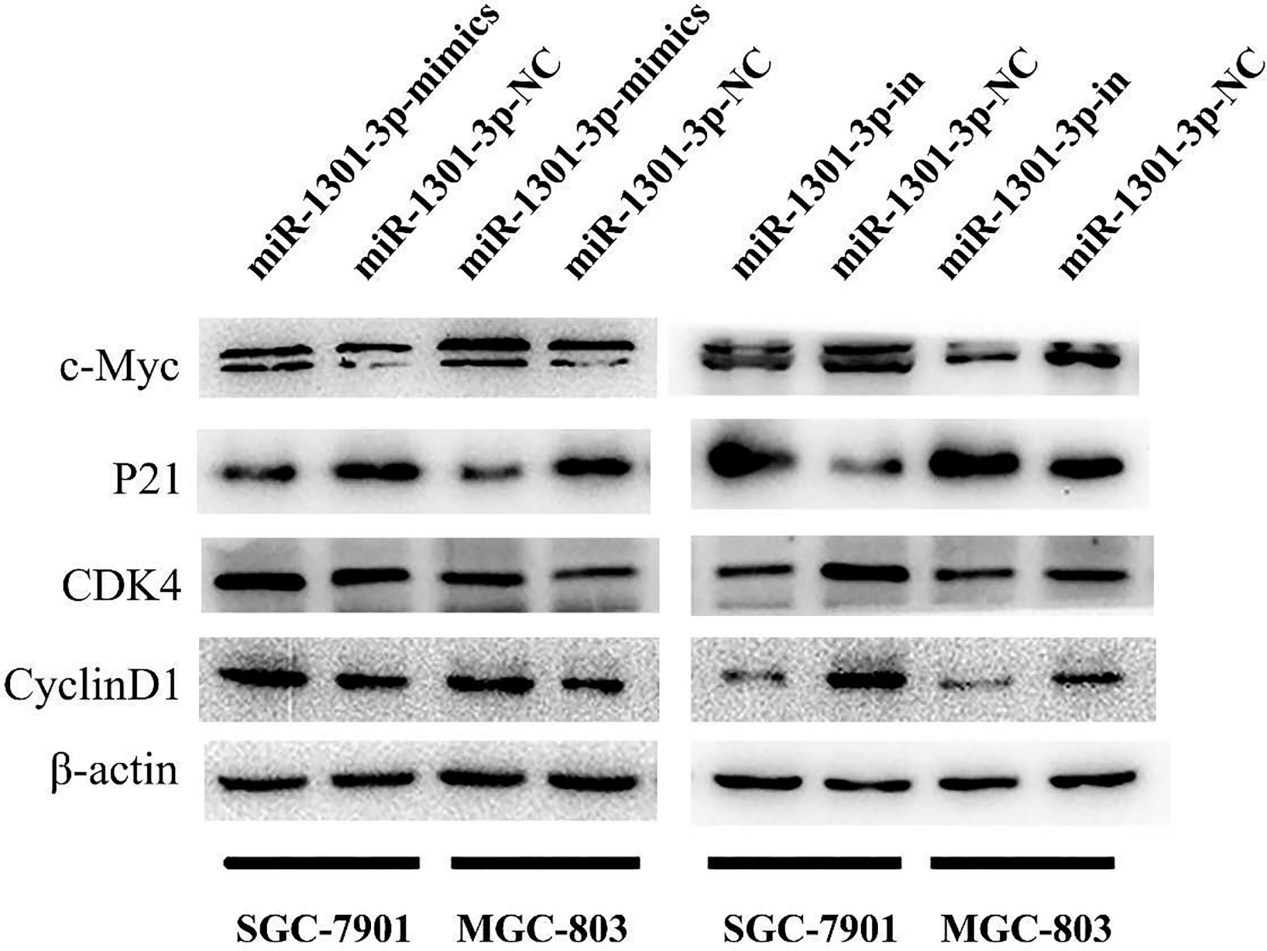
Expression of key proteins for G1/S transition. Overexpression of miR-1301-3p elevated the Cyclin D1, CDK4, c-Myc expression and inhibited P21 expression, knockdown of miR-1301-3p inhibited Cyclin D1, CDK4, c-Myc expression and elevated P21 expression.

To further figure out the underlying role of miR-1301-3p in GC, we carried out a gain-loss study. MGC-803 and SGC-7901 were treated with miR-1301-3p mimics, inhibitor as well as corresponding normal controls using lentivirus. qRT-PCR was utilized to verify the efficacy of transfection (**Figures 2A**, **3A**). Colony formation assay demonstrated upregulated miR-1301-3p could enhance GC cells proliferation, whereas knockdown of miR-1301-3p could suppress GC cells proliferation (**Figures 2B**, **3B**). Consistently, CCK-8 assay also suggested that upregulated miR-1301-3p promoted GC cells proliferation (**Figures 2C, D**), knockdown of miR-1301-3p inhibited GC cells proliferation (**Figures 3C, D**). To better elucidate the possible mechanism, on the basis of above results, we thereafter investigated the effects of miR-1301-3p on cell cycle. The cell cycle assay provided us with convincing results that compared with the control, overexpression of miR-1301-3p promoted cell cycle arrest in the G0/G1 phase and the number of cells in the S phase was increased in GC cells (**Figures 4A, B**). Oppositely, knockdown of miR-1301-3p had the adverse effects (**Figures 4C, D**). The key proteins for G1/S transition were also detected. Overexpression of miR-1301-3p elevated the Cyclin D1, CDK4, c-Myc expression and inhibited P21 expression while knockdown of miR-1301-3p inhibited Cyclin D1, CDK4, c-Myc expression and elevated P21 expression (**Figure 5**, **Figures S1A–D**), which suggested miR-1301-3p could promote the transformation of G1/S and accelerate cell cycle progression. Above results showed that overexpression of miR-1301-3p promoted GC cell proliferation by regulating cell cycle progression of GC *in vitro*.

### SIRT1 Was A Direct Target of miR-1301-3p in GC

**Figure 6:**
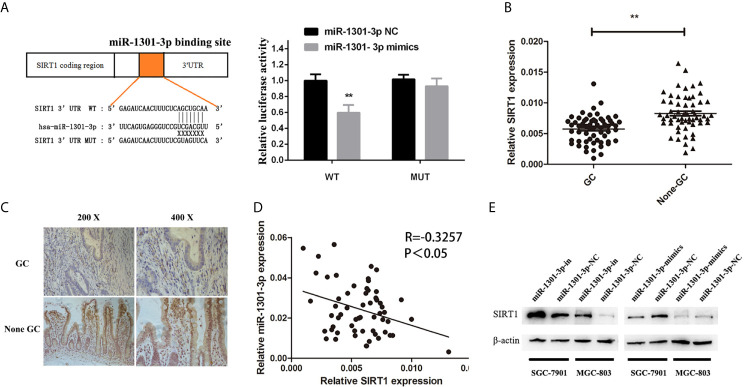
SIRT1 was a direct target of miR-1301-3p in GC. **(A)** Luciferase reporter assay proved that SIRT1 was a direct target of miR-1301-3p. **(B)** The expression levels of SIRT1 in 60 pairs of GC and adjacent normal tissues. **(C)** IHC investigations showed decreased SIRT1 expression in GC tissues compared with that in the paired adjacent normal tissues. **(D)** There was a negative correlation between the expression of miR-1301-3p and SIRT1 in GC specimens. **(E)** Overexpression of miR-1301-3p inhibited SIRT1 protein expression, knockdown of miR-1301-3p could promote SIRT1 protein expression. **p < 0.01.

**Figure 7:**
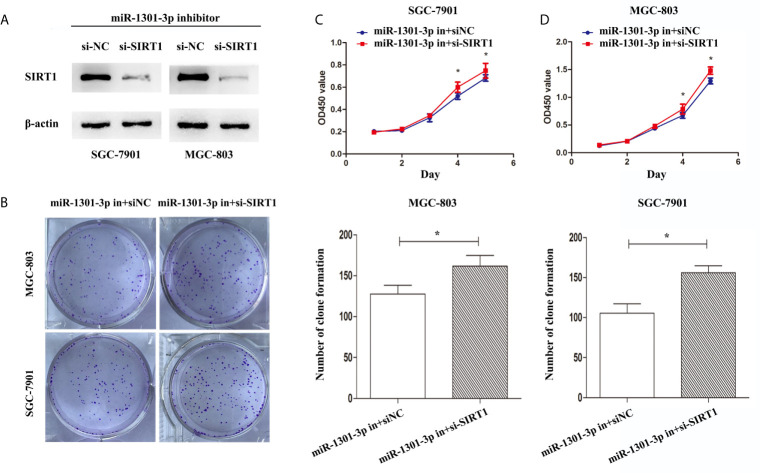
Knockdown of SIRT1 in gastric cancer cells transfected with miR-1301-3p inhibitor enhanced the ability of proliferation. **(A)** Western blotting was utilized to verify the efficacy of transfection. **(B)** Colony formation assay demonstrated that knockdown of SIRT1 and miR-1301-3p simultaneously promoted cell proliferation in gastric cancer. **(C, D)** CCK-8 assay demonstrated that knockdown of SIRT1 and miR-1301-3p simultaneously promoted cell proliferation in gastric cancer. *p < 0.05.

**Figure 8:**
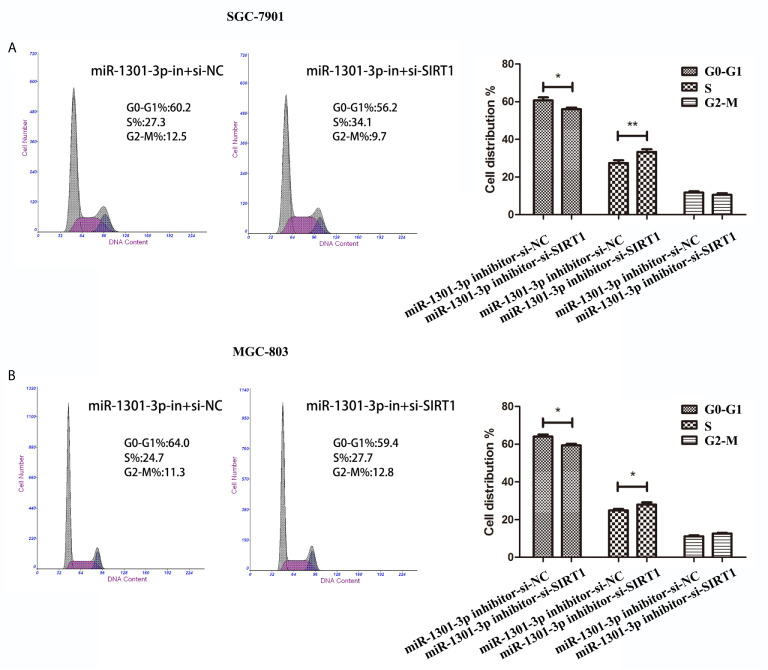
Cell cycle assay demonstrated that knockdown of SIRT1 and miR-1301-3p simultaneously promoted cell cycle progression. **(A, B)** Cell cycle assay in SGC-7901 and MGC-803 cells transfected with miR-1301-3p-in+si-NC and miR-1301-3p-in+si-SIRT1. *p < 0.05, **p < 0.01.

To more insightfully investigate the precise mechanism, we predicted the possible targets of miR-1301-3p on the basis of three databases including TargetMiner, microRNA.org, TarBase and identified that SIRT1 was one of the candidates of the target genes affected by miR-1301-3p. Bioinformatics analysis revealed one putative miR-1301-3p binding site in SIRT1 3’UTR. Luciferase reporter assay was used to verify the combination of miR-1301-3p and 3’UTR of SIRT1 mRNA. The 3’UTR region of SIRT1 with target and mutated sequences were cloned into the pMIR-REPORT luciferase reporter vector respectively. After co-transfecting pMIR-REPORT-SIRT1 and miR-1301-3p mimics, we detected a relatively reduced luciferase activity in HEK-293T cell, which showed that miR-1301-3p significantly reduced the relative luciferase activity of the wild-type SIRT1 3’UTR (**Figure 6A**). Furthermore, we detected the SIRT1 expression in GC and normal tissues by qRT-PCR. As shown in **Figure 6B**, SIRT1 was downregulated. IHC investigations also showed decreased SIRT1 expression in GC tissues (**Figure 6C**). Notably, miR-1301-3p and SIRT1 was negatively correlated in GC specimens (r=-0.3257, P<0.05) (**Figure 6D**). We evaluated the expression level of SIRT1 protein after lentiviral transfection by utilizing western blotting. We found that overexpressed miR-1301-3p could suppress SIRT1 protein expression whereas knockdown of miR-1301-3p could promote SIRT1 protein expression (**Figure 6E**, **Figures S1E, F**). We next downregulated SIRT1 using siRNA in SGC-7901 and MGC-803 cells, which had been verified by western blotting (**Figure 7A**, **Figure S1G**). Using colony formation assay, CCK-8 and cell cycle assay, we found that knockdown of SIRT1 could reverse the inhibition of GC cell proliferation (**Figures 7B–D**) and cell cycle progression (**Figure 8**, **Figure S2**) by miR-1301-3p inhibitor compared with control group. Taken together, these data suggested that SIRT1 was a direct target of miR-1301-3p.

### miR-1301-3p Facilitates Tumor Growth of GC Cells *In Vivo*

**Figure 9:**
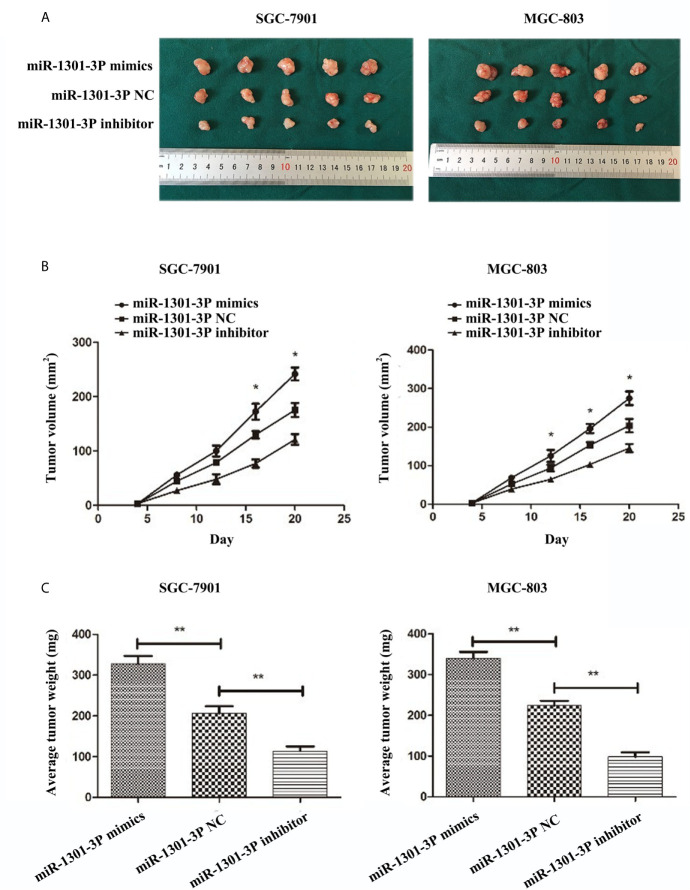
miR-1301-3p facilitates tumor growth of GC cells *in vivo*. **(A)** GC cells SGC-7901 and MGC-803 transfected with miR-1301-3p mimics facilitated the tumor formation in the flank of nude mice, GC cells SGC-7901 and MGC-803 transfected with miR-1301-3p inhibitor retarded the tumor formation in the flank of nude mice. **(B, C)** The graphs represented the growth tendency of tumors 3 weeks after inoculation. The volume and weight of tumors were calculated. *p < 0.05, **p < 0.01.

To evaluate the effects of miR-1301-3p on tumor growth *in vivo*, SGC-7901 and MGC-803 cells transfected with miR-1301-3p mimics, inhibitor and negative control lentivirus were injected subcutaneously into nude mice. The miR-1301-3p mimics group had a significant increase in tumor volume and weight compared with the control group. However, the miR-1301-3p-inhibitor group demonstrated the opposite effect (**Figure 9**).

## Discussion

In our study, we initially performed a comprehensive analysis of miRNA expression profiles and found miR-1301 was upregulated. To verify the above data, we evaluated the miR-1301 expression in GC and normal tissues. That miR-1301-3p promoted cell proliferation in GC could be demonstrated by CCK-8 assay and colony formation assay. Besides that, miR-1301-3p facilitated G1/S transition and resulted in deregulation of Cyclin D1, CDK4, c-Myc and P21 in GC cells. Meanwhile, experiments *in vivo* showed that miR-1301-3p could contribute to the growth of GC xenograft tumors. Further studies showed that the direct target of miR-1301-3p was SIRT1. These studies demonstrated the roles of miR-1301-3p in GC.

In recent years, short chain non-coding RNAs, especially miRNAs, and the study of their function, have greatly promoted our understanding of the development of tumors. Studies have shown that miRNAs play an important role on tumorigenesis and progression in many tumors, which suggested that miRNA could be used as tumor prevention, diagnosis and prognosis value of biomarkers or targets (20–26). For instance, a phase I clinical trial (NCT01829971) is ongoing to test miR-34 mimics that is encapsulated in lipid nanoparticles in hematological malignancies and several solid tumors (27). Depending on the specific functions of the targeted mRNA, miRNAs either function as cancer promoters or function as cancer suppressors in tumors (28–31). However, it has been well accepted that some miRNAs may play the role of tumor suppressor or oncogene by targeting multiple downstream genes in diverse tumor types (32, 33). For instance, miR-424-5p promotes GC proliferation by targeting Smad3 (34) and suppresses cervical cancer cell growth though decreasing the expression of KDM5B (35). miR-155 promotes hepatocellular carcinoma progression by suppressing PTEN through the PI3K/Akt pathway (36) and acts as a tumor suppressor by targeting CTHRC1 *in vitro* in colorectal cancer (37). miR-1301-3p acts as oncogene that accelerates the process of prostate carcinogenesis through targeting PPP2R2C (11) and anti-oncogene by inhibiting cell proliferation in glioma (12). However, the paradoxical results were reported in the progression of hepatic cancer (8–10). On the basis of our results, we speculate that there are tumor-specific transcription factors located upstream of miR-1301-3p in different tumor types, resulting in the differential expression of miR-1301-3p and its variable biological functions. The precise mechanisms need to be addressed in the future studies.

SIRT1, which belongs to the class III histone deacetylase family, is down-regulated in GC and leads to G1-phase arrest via NF-κB/cyclin D1 signaling, thereby inhibiting the proliferation of GC cells (19). In this study, SIRT1 was recognized as a direct target of miR-1301-3p. Overexpression of miR-1301-3p accelerated G1/S transition and led to deregulation of Cyclin D1, CDK4, c-Myc and P21 in GC cells. Previous studies have revealed the correlations between miRNAs and cell cycle (38). Induction of cell cycle progression is one of core mechanisms for tumor growth. Obviously, knockdown of SIRT1 and miR-1301-3p simultaneously promoted cell proliferation and accelerated cell cycle progression. These results demonstrated that miR-1301-3p promoted cell proliferation likely by targeting SIRT1 though cell cycle progression.

However, there are several limitations in our study. We did not overexpress SIRT1 in cells transfected with miR-1301-3p mimics to evaluate the role of SIRT1-mediated promotion in gastric carcinogenesis regulated by miR-1301-3p. In addition, the underlying signaling pathways participating in the regulation of miR-1301-3p were not investigated. Further study of gene expression profiles needs to be carried out to identify the activated signaling pathways.

In summary, this study provided convincing results that miR-1301-3p is upregulated and promotes cell proliferation by targeting SIRT1 in GC. As a result, miR-1301-3p may be a new treatment breakthrough for GC.

## Data Availability Statement

The original contributions presented in the study are included in the article/**Supplementary Material**. Further inquiries can be directed to the corresponding author.

## Ethics Statement

The Institutional Animal Care and Use Committee of Nanjing Medical University approve all animal experiments. Written informed consent was obtained from the individual(s) for the publication of any potentially identifiable images or data included in this article.

## Author Contributions

LY designed the experiments. DL, HF, XM, and CY performed the experiments. YH analyzed the data. YG, MJ, and ZX contributed reagents/materials/analysis tools. All authors contributed to the article and approved the submitted version.

## Funding 

This work was supported by National Natural Science Foundation of China (Grant/Award No. 81874219), the Natural Science Foundation of Jiangsu Province [Grant No. BK20171505], and Li yang City’s 2018 Annual research and development Plan Follows Nanjing Project (LC2019002).

## Conflict of Interest

The authors declare that the research was conducted in the absence of any commercial or financial relationships that could be construed as a potential conflict of interest.

## Supplementary Material

Supplementary Figure 1 **(A–D)**, Quantitative analysis of c-Myc, P21, CDK4, CyclinD1 protein levels in SGC7901 and MGC803 cells transfected with miR-1301-3p inhibitor, miR-1301-3p mimics and their control groups. **(E, F)**, Quantitative analysis of SIRT1 protein levels in SGC7901 and MGC803 cells transfected with miR-1301-3p inhibitor, miR-1301-3p mimics and their control groups. **(G)**, Quantitative analysis of SIRT1 protein levels in SGC7901 and MGC803 cells transfected with miR-1301-3p-in+si-NC and miR-1301-3p-in+si-SIRT1. *p < 0.05, **p < 0.01, ***p < 0.001.

Supplementary Figure 2 **(A, B)**, The protein expression of SIRT1, Cyclin D1, CDK4, c-Myc and P21 in SGC7901 and MGC803 cells transfected with miR-1301-3p-in+si-NC and miR-1301-3p-in+si-SIRT1. *p < 0.05, **p < 0.01, ***p < 0.001.
