# Supplementary figures and images for: miR-1301-3p Promotes Cell Proliferation and Facilitates Cell Cycle Progression via Targeting SIRT1 in Gastric Cancer
Source: Front Oncol. 2021 Apr 27;11:664242. doi: 10.3389/fonc.2021.664242 (PMC8112236; doi:10.3389/fonc.2021.664242)

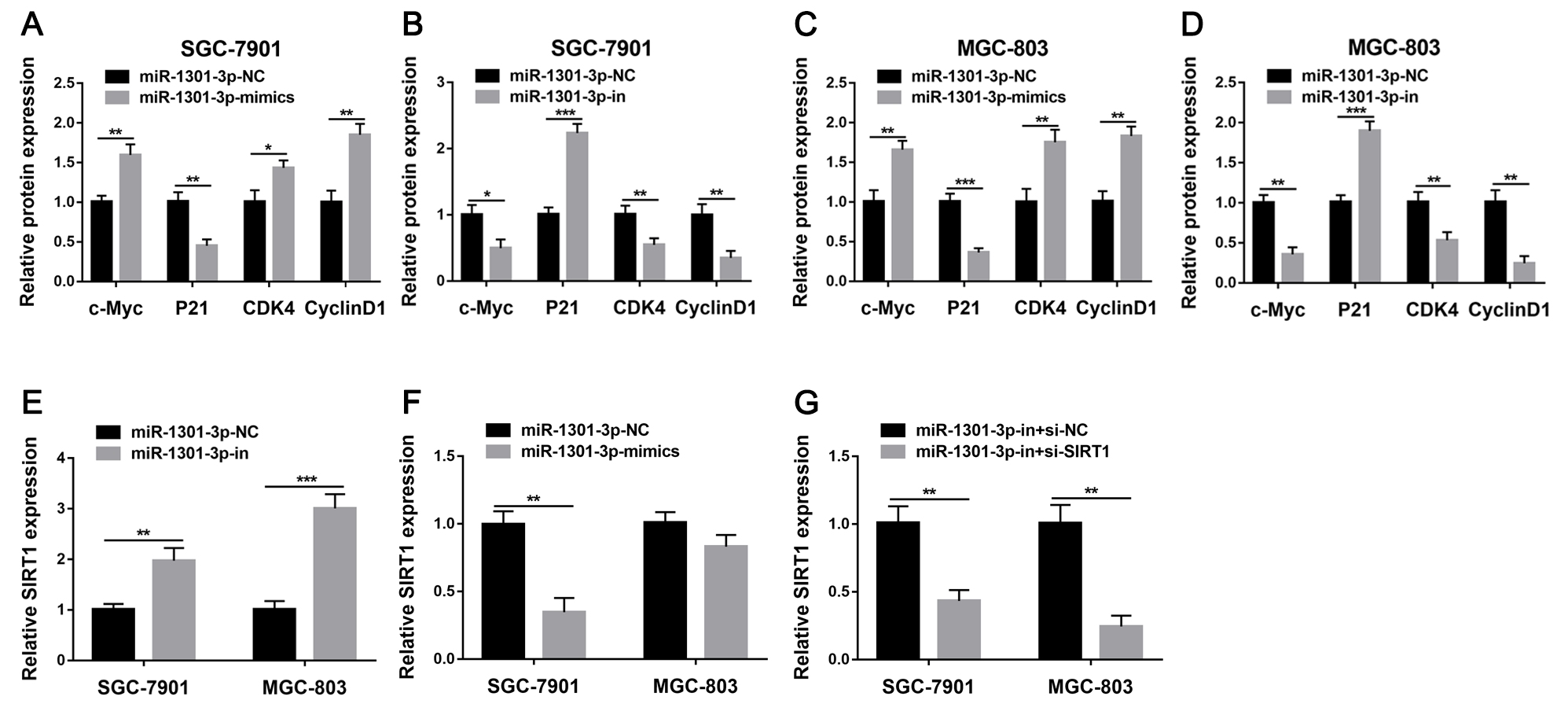

Supplement: Supplementary Figure 1 — (A–D), Quantitative analysis of c-Myc, P21, CDK4, CyclinD1 protein levels in SGC7901 and MGC803 cells transfected with miR-1301-3p inhibitor, miR-1301-3p mimics and their control groups. (E, F), Quantitative analysis of SIRT1 protein levels in SGC7901 and MGC803 cells transfected with miR-1301-3p inhibitor, miR-1301-3p mimics and their control groups. (G), Quantitative analysis of SIRT1 protein levels in SGC7901 and MGC803 cells transfected with miR-1301-3p-in+si-NC and miR-1301-3p-in+si-SIRT1. *p < 0.05, **p < 0.01, ***p < 0.001. [file Image_1.tif]

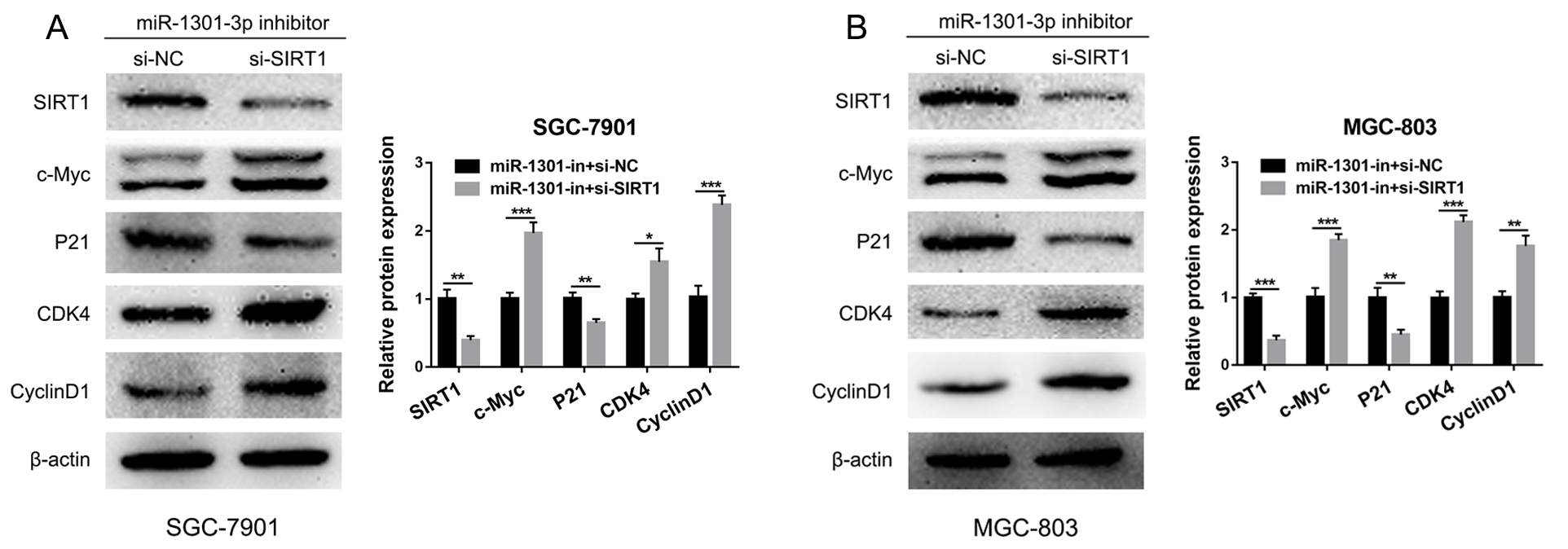

Supplement: Supplementary Figure 2 — (A, B), The protein expression of SIRT1, Cyclin D1, CDK4, c-Myc and P21 in SGC7901 and MGC803 cells transfected with miR-1301-3p-in+si-NC and miR-1301-3p-in+si-SIRT1. *p < 0.05, **p < 0.01, ***p < 0.001. [file Image_2.tif]
